# Detection of phase-dependent transcriptomic changes and Rubisco-mediated CO_2_ fixation into poly (3-hydroxybutyrate) under heterotrophic condition in *Ralstonia eutropha* H16 based on RNA-seq and gene deletion analyses

**DOI:** 10.1186/1471-2180-13-169

**Published:** 2013-07-23

**Authors:** Rie Shimizu, Kenta Chou, Izumi Orita, Yutaka Suzuki, Satoshi Nakamura, Toshiaki Fukui

**Affiliations:** 1 Department of Bioengineering, Graduate School of Bioscience and Biotechnology, Tokyo Institute of Technology, 4259 Nagatsuta, Midori-ku, Yokohama 226-8501, Japan; 2 Department of Medical Genome Science, the University of Tokyo, 5-1-5 Kashiwanoha, Kashiwa, Chiba 277-8562, Japan

**Keywords:** RNA-seq, *Ralstonia eutropha*, Polyhydroxyalkanoates, CO_2_ fixation, Cavin-benson-bassham cycle, Rubisco

## Abstract

**Background:**

*Ralstonia eutropha* H16 is well known to produce polyhydroxyalkanoates (PHAs), which are potential bio-based biodegradable plastics, in an efficient manner as an energy storage material under unbalanced growth conditions. To obtain further knowledge of PHA biosynthesis, this study performed a quantitative transcriptome analysis based on deep sequencing of the complementary DNA generated from the RNA (RNA-seq) of *R. eutropha* H16.

**Results:**

Total RNAs were extracted from *R. eutropha* cells in growth, PHA production, and stationary phases on fructose. rRNAs in the preparation were removed by repeated treatments with magnetic beads specific to bacterial rRNAs, and then the 36 bp sequences were determined using an Illumina high-throughput sequencer. The RNA-seq results indicated the induction of gene expression for transcription, translation, cell division, peptidoglycan biosynthesis, pilus and flagella assembly, energy conservation, and fatty acid biosynthesis in the growth phase; and the repression trends of genes involved in central metabolisms in the PHA production phase. Interestingly, the transcription of genes for Calvin-Benson-Bassham (CBB) cycle and several genes for β-oxidation were significantly induced in the PHA production phase even when the cells were grown on fructose. Moreover, incorporation of ^13^C was observed in poly(3-hydroxybutyrate) synthesized by *R. eutropha* H16 from fructose in the presence of NaH^13^CO_3_, and further gene deletion analyses revealed that both of the two ribulose 1,5-bisphosphate carboxylase (Rubiscos) in CBB cycle were actually functional in CO_2_ fixation under the heterotrophic condition.

**Conclusions:**

The results revealed the phase-dependent transcriptomic changes and a CO_2_ fixation capability under heterotrophic conditions by PHA-producing *R. eutropha*.

## Background

*Ralstonia eutropha* H16, a Gram-negative facultative chemolithoautotrophic bacterium, can utilize various organic compounds such as sugars, organic acids, fatty acids, and plant oils in the heterotrophic growth mode, while in the absence of organic substrates, it thrives autotrophically on H_2_ and CO_2_ as the energy and carbon sources, respectively, where CO_2_ is fixed by Calvin-Benson-Bassham (CBB) cycle [1]. This strain has been also known to accumulate poly(3-hydroxybutylate) [P(3HB)] as a storage compound under unbalanced growth conditions, if a carbon source is available in excess while another essential element (N, O, P, S, or metals) is growth limiting at the same time. It has been estimated that P(3HB) accumulation has a role in survival under the stress conditions.

Bacterial P(3HB) has attracted industrial attention because it is a biodegradable thermoplastic that can be produced from renewable carbon sources; thus it is a possible alternative to petroleum-based polymer materials. A number of studies have focused on P(3HB) biosynthesis by *R. eutropha* H16, particularly regarding the biosynthetic pathways and enzymes, as well as the biogenesis, structure, and mobilization of intracellular P(3HB) granule [2-7]. In this strain, P(3HB) is synthesized from the central intermediate acetyl-CoA through three step reactions catalyzed by β-ketothiolase (PhaA), NADPH-dependent acetoacetyl-CoA reductase (PhaB1), and PHA synthase (PhaC1), the genes of which are clustered in *phaC1-A-B1*. The intracellular P(3HB) exists as granules coated with a layer of phospholipids and several proteins, *i. e.* PhaC1, P(3HB) depolymerases (PhaZs) and phasins (PhaPs). The *phaC1-A-B1* operon or the respective genes from *R. eutropha* H16 have been used to confer the capability for P(3HB) biosynthesis to non-PHA-producing bacteria such as *Escherichia coli*, as well as higher plants [8]. This strain has also been used as a host for metabolic engineering with the aim of biosynthesizing PHA copolyesters with more flexible properties compared with the brittle and hard P(3HB) homopolymer [9-15].

The complete genome analysis of *R. eutropha* H16 was reported in 2006 [16]. The genome consists of three circular replicons; chromosome 1 (4.05 Mbp), chromosome 2 (2.91 Mbp), and megaplasmid pHG1 (0.45 Mbp); and the genes for essential metabolisms and cellular functions are located on chromosome 1. The genome information has facilitated the genome-wide transcriptome analysis of this strain. Hitherto, transcriptome analyses of *R. eutropha* were performed using a DNA microarray technique. Peplinski *et al*. reported a comparison of the transcriptomes of wild-type strain H16 and the two PHA-negative strains in different growth phases based on competitive hybridization [17]. They observed significant differences in the transcription levels of a large number of genes in these strains, including genes involved in lipid metabolisms. However, the comparison of transcriptomes in the exponential growth and P(3HB) biosynthesis phases of *R. eutropha* was unclear. Brigham *et al*. carried out a transcriptomic comparison of *R. eutropha* H16 cells grown in fructose- and trioleate-containing media, and identified two gene clusters responsible for β-oxidation [18].

Hybridization-based DNA microarray methods have mainly been used for global transcriptome analysis; however, these methods exhibit a relatively low dynamic range for detecting transcription because of two reasons. One is a high level of noise caused by cross-hybridization, and the other is saturation and poor sensitivity at very high and low transcriptional levels, respectively [19]. Recently, the direct sequencing of complementary DNA generated from RNA (RNA-seq) based on high-throughput DNA sequencing technology was often used to study RNA population within the cells [20]. Many studies have demonstrated that RNA-seq has several advantages over the previous microarray methods used for transcriptional analysis, including a larger dynamic range, lower background noise, and greater sensitivity [21]. In addition, this technique enables comparison of the transcription levels of different genes in the same sample. Although RNA-seq was initially difficult to apply to bacterial cells without poly-A tails in their mRNA, enrichment of the mRNA by rRNA pulldown and great improvement in the sequencing depth of the recent sequencer can overcome this problem [21].

In this study, we applied RNA-seq to profile and quantify the transcription levels of *R. eutropha* H16 genes in the growth, PHA biosynthesis, and stationary phases on fructose. We successfully detected a number of interesting transcriptomic changes that depended on the cellular phases. Recently, Brigham *et al*. carried out a microarray analysis of this strain in different phases, and identified the regulation of PHA biosynthesis by a stringent response [22]. Several of our results were consistent with those based on the microarray analysis as described below, and one of the interesting results was a significant induction of CBB cycle in the PHA production phase on fructose. Thus, we investigated the possibility of CO_2_ fixation during P(3HB) biosynthesis by *R. eutropha* H16 under heterotrophic conditions, and demonstrated that both of the two ribulose 1,5-bisphosphate carboxylase (Rubisco) in the transcriptionally activated CBB cycle actually played a role in incorporation of ^13^C into P(3HB) synthesized from fructose in the presence of NaH^13^CO_3_.

## Result and discussion

### Cultivation, sample preparation, and RNA sequencing

**Figure 1:**
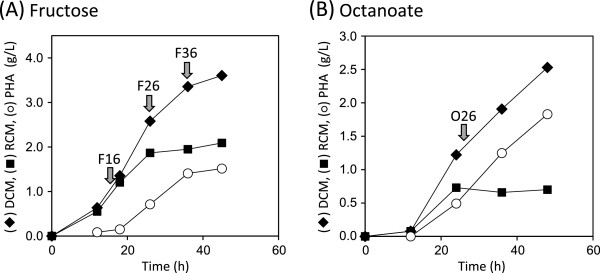
**Growth and PHB biosynthesis properties of *R. eutropha *H16.** The cells were cultivated in a mineral salts medium containing 0.2% NH_4_Cl and 2.0% (w/v) fructose **(A)** or 0.1% x5 (w/v) sodium octanoate **(B)**. Allows indicate the time point at which samples were withdrawn. F16, exponential growth phase on fructose; F26, PHA production phase on fructose; F36, stationary phase on fructose; O26, PHA production phase on octanoate. DCM, dry cell mass; RCM, residual cell mass. (This figure is the same as that in Ref. [23]).

*R. eutropha* H16 was cultivated in a mineral salt medium containing 0.2% (w/v) NH_4_Cl to separate the PHA production phase from the growth phase precisely. As shown in Figure 1(A), the cells grew initially without PHA biosynthesis and started to accumulate P(3HB) after 18 h of cultivation. P(3HB) was produced up to 42 wt% of dry cell mass during 26–36 h with a nearly constant residual cell mass, and then reached to stationary. Total RNA was isolated from cells in the growth phase at 16 h (referred to as F16), PHA production phase at 26 h (F26), and stationary phase at 36 h (F36) [Figure 1(A)]. When octanoate was supplied as a non-sugar growth substrate, the cell growth and PHA biosynthesis initially occurred simultaneously and further PHA production was observed after the saturation of cell growth [Figure 1(B)]. Therefore, the total RNA was isolated from cells in the PHA production phase not associated with cell growth at 26 h (O26), 2 h after the third stepwise addition of octanoate.

The rRNA in the total RNA was removed repeatedly, and the enriched mRNA was subjected to RNA-seq with two technical replicates. The numbers of mapped reads (36 bp) with no mismatches reached about 26–43 million reads per run (Table 1). Despite the removal of rRNA twice, 72–89% of the reads still mapped to rRNA regions, which indicated that the mRNA enrichment procedure required further optimization. The reads that mapped onto *rrn* operons (consisting of *rrs*, tRNA-Ile, tRNA-Ala, *rrl*, and *rrf*) were discarded from the set of reads, and the remaining reads were used as the total reads. We obtained 3–10 million reads other than *rrn* operons that mapped onto the *R. eutropha* genome, which were considered to be sufficient for transcriptome analysis of the small bacterial genome. The genes with significant changes in expression were used in the subsequent analysis (*P* < 0.05), *i. e.* 5,553 genes out of a total 6,635 genes. Of the statistically non-significant genes, over 90% of the genes were silent or had weak expression with reads per kilobase per million mapped reads (RPKM) values of <250 in all of the samples examined.

**Table 1:** Summary of RNA-sequencing

**Sample**	**Run**	**F16**	**F26**	**F36**	**O26**
C-source, cultivation time		Fructose, 16 h	Fructose, 26 h	Fructose, 36 h	Octanoate, 26 h
Cellular phase		Growth	PHA production	Stationary	PHA production
Number of reads	1	38,214,032	39,930,488	33,340,159	26,442,674
	2	41,400,577	40,262,640	28,544,022	40,339,722
Reads on rRNA regions^a^	1	27,521,315	34,573,643	27,787,869	23,463,979
	2	30,737,637	34,721,392	23,478,331	35,483,825
Reads on CDSs other than rRNA regions^a^ (Total reads)	1	10,692,717	5,356,845	5,552,290	2,978,695
	2	10,662,940	5,541,248	5,065,691	4,855,897

^a^H16_A1659-A1661 (*rrsA-rrlA-rrfA*), H16_A3306-A3302 (*rrsB*-tRNA[Ile]-tRNA[Ala]-*rrlB*-*rrfB*), and H16_A3513-A3509 (*rrsC*-tRNA[Ile]-tRNA[Ala]-*rrlC*-*rrfC*) on chromosome 1, and H16_B0151-B0155 (*rrsD*-tRNA[Ile]-tRNA[Ala]-*rrlD*-*rrfD*) and H16_B0769-B0773 (*rrsE*-tRNA[Ile]-tRNA[Ala]-*rrlE*-*rrfE*) on chromosome 2.

### Overview of *R. eutropha* transcriptomes

**Figure 2:**
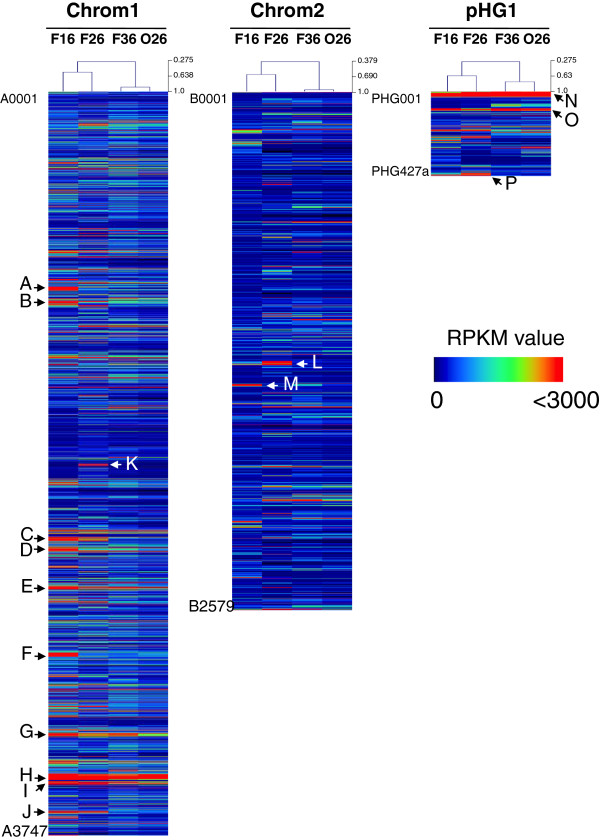
**Heatmaps of transcriptomes in *R. eutropha *H16 in different phases.** The expression pattern is shown by the color scale based on RPKM value of each gene on chromosome 1 (left), chromosome 2 (center), and pHG1 (right), except for rRNA- and tRNA-coding genes and non-significant genes in expression (P > 0.05). The arrows A-P indicate highly expressed clusters.

Clustering of the four transcriptomes (F16, F26, F36, and O26) based on the calculated RPKM values detected global changes in the transcription levels of a number of genes, which depended on the cellular phases (Figure 2). However, the clustering analysis indicated the strong resemblance of the O26 transcriptome to that of F36. In particular, there are almost no significant differences between F36 and O26 in terms of the expression levels of genes encoding β-oxidation enzymes, including the two gene clusters previously identified by Brigham *et al*. [18]. These facts implied that the transcriptional changes related to fatty acid metabolism had already fulfilled 2 h after the stepwise addition of octanoate at 24 h. Thus, the O26 transcriptome was not examined in detail in the present study. Further optimization of the time point for RNA isolation should be considered to obtain the transcriptomes of *R. eutropha* grown on fatty acids. The medium-chain-length (mcl)-(*R*)-3-hydroxyacyl-CoA molecules are provided through β-oxidation in several PHA-producing bacteria, including *R. eutropha*[9,11-14,24], therefore, the transcriptomic changes that depended on the chain length of fatty acids would be valuable information.

Table 2 summarizes highly expressed gene clusters during cultivation on fructose (indicated by arrows in Figure 2). Gene clusters that encoded a number of ribosomal proteins and RNA polymerase subunits (H16_A3457-A3484 and H16_A3490-A3505), and membrane-bound hydrogenase subunits along with the accessory proteins (PHG001-PHG023) were highly expressed throughout cultivation. In the growth phase (F16), the expression of gene clusters related to pilus assembly proteins (H16_A0981-A0987), cell-division and peptidoglycan biosynthesis (H16_A3268-A3282), fatty acid biosynthesis (H16_A2560-A2572), ATP synthase subunits (H16_A3636-A3643), and NADH dehydrogenase subunits (H16_A1050-A1063) were high, which are consistent with their importance of cell assembly and energy conservation for cell growth. Interestingly, significant transcriptional induction in the PHA production phase (F26) was observed for the gene clusters H16_A1949-A1957, H16_B1380-B1395 and PHG416-PHG427, of which the latter two clusters contained *cbb* operons that encode CBB cycle enzymes involved in CO_2_ fixation (see below).

**Table 2:** **Highly transcribed clusters in *R. eutropha *H16 during cultivation on fructose**

**Clusters**^**a**^	**Gene IDs**	**Representative products or functions**	**Highly transcribed phase(s)**
A	H16_A0976-A0993	Pilus assembly proteins	Growth
B	H16_A1047-A1063	NADH dehydrogenase subunits, triosephosphate isomerase TpiA	Growth
C	H16_A2305-A2321	Translation initiation factor InfB, transcription elongation factor NusA, cytchrome c oxisdase subunits	Growth
D	H16_A2359-A2369	RNA-binding protein Hfq, GTP-binding protein EngA, histidyl-tRNA synthetase, nucleoside diphosphate kinase	Growth
E	H16_A2560-A2572	Sigma factor RpoE, sigma E-negative regulatory proteins, fatty acid biosynthesis	Growth
F	H16_A2889-A2905	Cell wall biogenesis	Growth
G	H16_A3268-A3282	Cell division proteins, peptidoglycan biosynthesis	Growth
H	H16_A3457-A3484	Ribosomal proteins, RNA polymerase subunit α, translation initiation factor InfA	Growth, PHA production, Stationary
I	H16_A3490-A3505	Ribosomal proteins, elongation factors, RNA polymerase subunits ββ’, transcription antiterminator NusG	Growth, PHA production, Stationary
J	H16_A3636-A3643	F_0_F_1_ ATP synthase subunits	Growth
K	H16_A1949-A1957	Metylmalonyl-CoA mutase, K^+^ transport flavoprotein	PHA production
L	H16_B1380-B1395	Calvin-Benson-Bassham cycle	PHA production
M	H16_B1497-B1503	ABC-type fructose transporter, Entner-Doudoroff pathway	Growth
N	PHG001-PHG023	Membrane-bound hydrogenase subunits, hydrogenase accessory proteins	Growth, PHA production, Stationary
O	PHG088-PHG096	Soluble hydrogenase subunits, hydrogenase accessory proteins	Growth, Stationary
P	PHG416-PHG427	Calvin-Benson-Bassham cycle	PHA production

^**a**^Indicated in Figure 2.

The highly expressed genes with RPKM values >20,000 in at least one of the three phases in the fructose-containing medium are shown in Additional file 1: Table S1. A number of ribosomal protein genes were well expressed in the growth phase, as well as several transcription and translation factors, *groES-EL* (H16_A0705-A0706), *secY* and *secE* (H16_A3464 and H16_A3503), and such others. The high-level expression of *rpoN* (H16_A0386) was observed throughout cultivation, which was particularly high in the nitrogen-deficient PHA production phase as expected. H16_A3402, which encodes an outer membrane protein (porin), exhibited the highest RPKM values in F16 and F26; while H16_B2205, which encodes a cold shock protein, exhibited high-level expression throughout cultivation on fructose, particularly in F16.

Additional file 1: Tables S2 and S3 show the highly up-regulated and down-regulated genes in the PHA production phase to the growth phase (F26/F16), respectively. The highly down-regulated genes, *i. e.* genes with high induction in the growth phase, included *flg* cluster (H16_B0258-B0271) and two *fli* clusters (H16_B0561-B0567 and H16_B2360-B2373) related to flagella assembly, as well as several genes in *che* operon (H16_B0229-B0245) that are related to chemotaxis (Additional file 1: Table S3). Raberg *et al*. reported that flagellation was strongly occurred during growth and stagnated during PHA biosynthesis [25]. Similar results were obtained in a previous microarray-based comparison of *R. eutropha* H16 and a PHA-negative mutant PHB^-^4 [17].

A recent microarray analysis by Brigham *et al*. reported that PHB production was regulated by a stringent response, because most of the upstream regions of the strongly up-regulated genes during nitrogen stress contained the consensus elements for σ^54^-family promoters [22]. Many of the genes were also highly up-regulated by 20–50 fold during the nitrogen-depleted PHA production phase in the present study, such as H16_A0359, H16_A2801, H16_B0780, H16_B0948, and H16_B1156 (Additional file 1: Table S2). A gene cluster that encodes potential nitrogen-scavenging transporters and enzymes (H16_A1075-A1087) was also up-regulated in F26 by 4–16 fold to F16 (data not shown). The expression ratios were much less than 50-491-fold detected in the microarray analysis [22], but the present RNA-seq analysis supported the expression regulation for these genes by the stringent response.

### Transcriptome changes related to major metabolic processes and cellular functions

#### Sugar degradation

**Figure 3:**
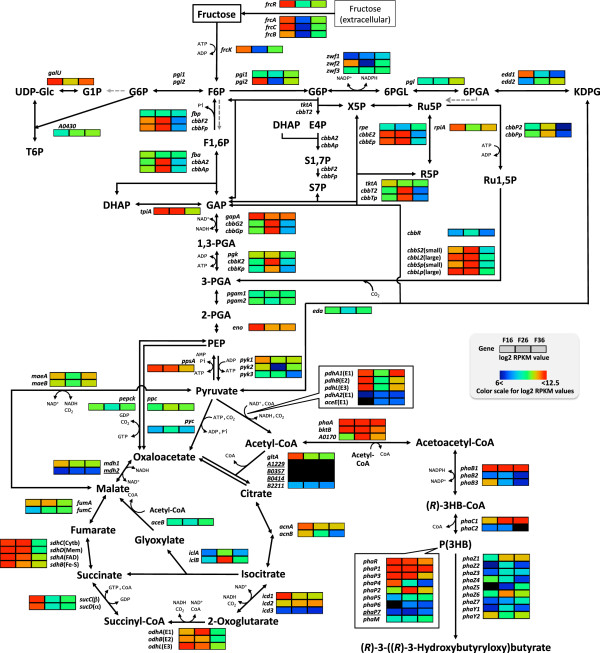
**Expression levels of genes involved in central metabolisms including PHA metabolism in *R. eutropha *H16 at growth phase F16, PHA production phase F26, and stationary phase F36 on fructose.** The log2-transformed RPKM values are visualized using the rainbow color scale in the figure. Genes with the *P* value above the threshold (*P* > 0.05) are underlined. Abbreviations: F6P, fructose-6-phosphate; F1,6P, fructose-1,6-bisphosphate; G1P, glucose-1-phosphate; G6P, glucose-6-phosphate; 6PGL, 6-phospho-1,5-gluconolactone; 6PGA, 6-phosphogluconate; KDPG, 2-keto-3-deoxygluconate-6-phosphate; UDP-Glc, UDP-glucose; T6P, trehalose-6-phosphate; X5P, xylose-5-phosphate; Ru5P, ribulose-5-phosphate; R5P, ribose-5-phosphate; Ru1,5P, ribulose-1,5-bisphosphate; E4P, erythrose-4-phosphate; S1,7P, sedoheptulose-1,7-bisphosphate; S7P, sedoheptulose-7-phosphate; GAP, glyceraldehyde-3-phosphate; DHAP, dihydroxyacetone phosphate; 1,3-PGA, 1,3-diphsphoglycerate; 3-PGA, 3-phosphoglycerate; 2-PGA, 2-phosphoglycerate; PEP, phosphoenolpyruvate; (*R*)-3HB-CoA, (*R*)-3-hydroxybutyryl-CoA; P(3HB), poly((*R*)-3-hydroxybutyrate); cyt b, cytochrom *b* subunit; mem, membrane anchor subunit; FAD, flavoprotein subunit; Fe-S, iron-sulfur subunit.

The genome analysis of *R. eutropha* H16 has identified three important clusters participated in fructose degradation in chromosome 2. The genes in cluster 1 (H16_B1497-B1503), which are *frcRACBK*, *pgi2*, and *zwf2* were significantly induced in the growth phase (Figure 3), suggesting the important roles in transportation and conversion of extracellular fructose to 6-phosphogluconolactone for growth. The genes in cluster 2, which are *glk*, *zwf3*, *pgl*, and *edd2* (H16_B2564-B2567) have roles in sugar phosphorylation and Entner-Doudoroff (ED) pathway. The expression levels of these genes were low in F16 and F26, and slightly increased in F36. The cluster 3 (H16_B1211-B1213), which consists of a gene of putative 2-amino-2-deoxy-D-gluconate hydrolase and *kdgK* for glucosaminate degradation, and *eda* involved in ED pathway, was observed to be induced in the growth phase.

*R. eutropha* H16 lacks a gene encoding phosphofructokinase [16]; therefore, Embden-Meyerhof (EM) pathway is assumed to function in conversion of glyceraldehyde 3-phosphate generated via ED pathway into pyruvate, or in gluconeogenesis on non-sugar growth substrates. Of the genes for EM pathway, transcription of *gapA* (H16_A3146) and *eno* (H16_A1188) were extensively high in F16. While, reaction steps between 3-phosphoglycerate and fructose-6-phosphate in EM pathway are shared with CBB cycle. *R. eutropha* possesses two sets of CBB enzymes, which are encoded in *cbb*_*c*_ and *cbb*_*p*_ operons in chromosome 2 (H16_B1383-B1395) and megaplasmid pHG1 (PHG416-PHG427), respectively [1]. We observed that the two *cbb* operons were expressed in the growth phase at levels comparable to the genes for ED pathway. Pentose-phosphate cycle is thought to be defective in *R. eutropha* H16 because of a lack of 6-phosphogluconate dehydrogenase; therefore, several enzymes in CBB cycle would have a role in establishing the non-oxidative branch of pentose-phosphate cycle, along with *rpe* (H16_A3317), *rpiA* (H16_A2345), *tal* (H16_A2346), *tktA* (H16_A3147) located in loci other than the *cbb* operons, thereby providing essential precursors for nucleotides and aromatic amino acids. The expression of *cbb* genes has been known to be activated by a transcriptional regulator CbbR (H16_B1396), which is encoded within *cbb*_*c*_ operon [26] when the intracellular concentration of phosphoenolpyruvate (PEP) becomes low under autotrophic conditions [27]. Several studies have also reported that partial derepression of the *cbb* genes also occurred during the late growth phase on a few substrates, such as fructose and gluconate [1,28,29]. Examination of our RNA-seq revealed that, even with partial derepression, the induction of both of the *cbb* operons in F26 was significant differences between the transcriptomes in the growth and PHA production phases, as shown in Figures 2 and 3. The previous microarray analyses of *R. eutropha* also showed that the transcription levels of the *cbb* operons were higher in the wild-type H16 strain than in a PHA-negative mutant PHB^-^4 [17], and they were induced in the absence of nitrogen [22]. PHA biosynthesis from acetyl-CoA may accompany a reduction in the intracellular concentration of PEP, which could lead to the transcriptional activation of the *cbb* operons.

#### Pyruvate metabolisms and TCA cycle

E1, E2, and E3 are components of the pyruvate dehydrogenase complex, which are encoded by *pdhA1* (H16_A1374), *pdhB* (H16_A1375), and *pdhL* (H16_A1377), respectively, and they were highly induced in the growth phase. In particular, *pdhL* exhibited an 18.5-fold increased expression in the growth phase compared with the PHA production phase, which was consistent with a previous observation that disruption of *pdhL* decreased the growth rate and PHA productivity on fructose [30]. *pdhA2* (H16_A1753) and *aceE* (H16_B1300), which encode paralogs of PdhA1 and PdhL, respectively, were barely expressed throughout cultivation. *gltA* (H16_A2627), *acnA* and *acnB* (H16_A2638 and H16_B0568, respectively), and *icd1* and *icd2* (H16_A3056 and H16_B1931, respectively), which encode enzymes for the conversion of C_6_-acids in TCA cycle, were highly expressed in the growth phase, but had slightly lower expression levels in the PHA production and stationary phases, except for the constitutively transcribed *icd2*. In addition to *gltA*, four genes are related to citrate synthase in *R. eutropha* H16, but we observed weak expression of H16_B2211 and negligible expression of the other three genes. The genes that encode other TCA cycle members also exhibited variable expression. For example, *odhABL* (H16_A2325-A2323) and *sdhCDAB* (H16_A2632-A2629) tended to be highly expressed in the growth and PHA production phases, whereas *sucCD* (H16_A0547-A0548) were induced in the growth phase. The genes for methylcitrate pathway [31] were constitutively expressed, although the level of expressions were very weak during the cultivation on fructose.

*iclA* (H16_A2211) and *iclB* (H16_A2227), both encodes isocitrate lyase in glyoxylate bypass, were observed to be highly induced in the PHA production phase. In particular, the transcription of *iclB* in F26 increased 33-fold as compared to that in F16. This result suggested a drastic change in the carbon flux from TCA cycle to glyoxylate bypass during PHA biosynthesis, but Brigham *et al*. have demonstrated that single disruptions of *iclA* or *iclB* did not affect the growth and PHA biosynthesis in *R. eutropha* H16 grown on fructose [18]. *pyc* (H16_A1251), *pepck* (H16_A3711) and *ppc* (H16_A2921) were present in the genome as genes encoding potential enzymes related to anaplerotic formation of oxaloacetate. A previous study reported that transcription and enzyme activities were detected only for *pepck* among the three genes in *R. eutropha*[32], whereas the present RNA-seq results indicated moderate expression of *ppc* and *pepck* as well as weak but actual expression of *pyc* throughout cultivation.

In *Escherichia coli* during high-cell density cultivation, it has been reported that genes involved in EM pathway and pentose-phosphate cycle were highly expressed in the stationary phase, probably to compensate for the reduced TCA cycle activity [33]. The transcription of genes for ED pathway in *Zymomonas mobilis* significantly increased under anaerobic ethanol-producing conditions to facilitate energy conservation [34]. In *R. eutropha* under the PHA biosynthesis condition, we observed a decreasing trend in expression of the genes in ED pathway and TCA cycle. The activity of ED pathway and TCA cycle during the PHA production phase is probably attributable to pre-existing as well as newly synthesized enzymes with the reduced transcription. The probably decreased flux of central metabolisms were supported by our recent metabolomics analysis of *R. eutropha* H16 that detected lower intracellular concentrations of many sugar phosphates in the PHA production phase than in the growth phase on fructose [23]. It can be assumed that the decreased metabolic activity appeared to be enough to maintain cellular viability and P(3HB) synthesis in a condition not associated with cell growth, as seen in *Corynebacterium glutamicum* in a glutamate-producing condition [35].

#### *de novo* Fatty acid synthesis and β-oxidation

**Figure 4:**
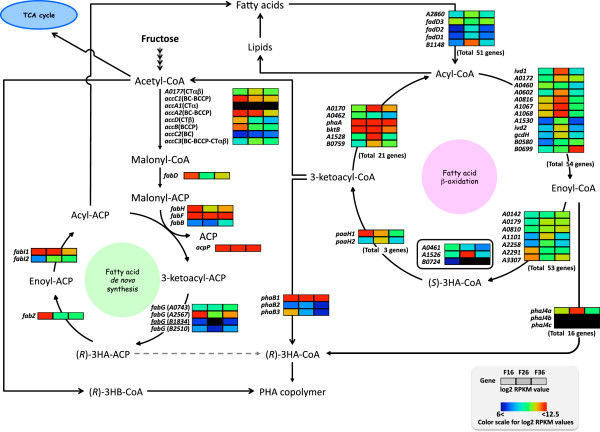
**Transcription levels of genes involved in fatty acid biosynthesis and β-oxidation in *R. eutropha *H16 at growth phase F16, PHA production phase F26, and stationary phase F36 on fructose.** With respect to β-oxidation enzymes, selected genes of which specific name has been assigned, or RPKM value are larger than 1,000 at least one of the three phases are shown. The log2-transformed RPKM values are visualized using the rainbow color scale in the figure. Genes with the *P* value above the threshold (*P* > 0.05) are underlined. Abbreviations: ACP, acyl-carrier protein; BC, biotin carboxylase; CT, carboxyl transferase; BCCP, biotin carboxyl carrier protein; (*R*)-3HB-CoA, (*R*)-3-hydroxybutyryl-CoA; (*R*)-3HA-CoA, (*R*)-3-hydroxyacyl-CoA; (*R*)-3HA-ACP, (*R*)-3-hydroxyacyl-acyl carrier protein. A0461, A1526, and B0724 are genes for putative β-oxidation multifunctional enzymes.

In* R. eutropha* H16, *accA1*, *accA2*, *accB*, *accC1*, *accC2*, *accC3*, and *accD* have been annotated as genes of the acetyl-CoA carboxylase (ACC) subunits. Based on a consideration of the general quaternary structure of ACC and the expression levels of these genes, the major ACC in this strain probably consisted of AccA1 (H16_A1223) as the carboxyl transferase subunit α (CTα), AccD (H16_A2611) as the carboxyl transferase subunit β (CTβ), AccB and AccC2 (H16_A3171-A3172) as the biotin carboxyl carrier protein (BCCP) and biotin carboxylase (BC), respectively. The expression levels of these genes were high in the growth phase, and then slightly decreased in the PHA production phase (Figure 4). *accC1* (H16_A0184, BC-BCCP) and H16_A0177 (CTαβ) may be another pair of ACC or the related carboxylase, because these had weak and similar expression behaviors to each other. The expression levels of *accA2* (H16_A2142, BC-BCCP) and *accC3* (H16_A3290, BC-BCCP-CTαβ) were negligible throughout cultivation on fructose. The genes *fabHDG*-*acpP*-*fabF* (H16_A2569-A2565), *fabZ* (H16_A2044), and *fabI1* (H16_A2410), which are involved in *de novo* fatty acid biosynthesis, were highly expressed in the growth phase, but many of the genes still had rather high expression levels in the PHA production phase.

A high number of genes in *R. eutropha* H16 are annotated as enzymes that potentially functions in fatty acid β-oxidation, which indicates the possible versatility of this strain for degradation of various hydrophobic compounds. Based on a detailed domain search, we identified 51 genes for acyl-CoA synthetase (ACS), 54 genes for acyl-CoA dehydrogenase (ACDH), 53 genes for enoyl-CoA hydratase (ECH), 3 genes for 3-hydroxyacyl-CoA dehydrogenase (3HCDH), and 21 genes for β-ketothiolase (KT). In fact, our RNA-seq examination revealed that many genes for putative β-oxidation enzymes were even expressed on fructose, as shown in Figure 4. The previous microarray study revealed that the two gene clusters of H16_A0459-A0464 and H16_A1526-A1531 were induced and in deed played important roles during β-oxidation in the cells grown on trioleate [18]. It was observed that the cluster H16_A0459-A0464 (which contains ACDH, 3HCDH-ECH fusion, KT, and ECH) was expressed weakly throughout cultivation on fructose, while the cluster H16_A1526-A1531 (which contains ECH-3HCDH fusion, KT, and ACDH) exhibited approximately 8.5 to 11.4-fold increased expression in the PHA production phase compared with that in the growth phase. *fadD3* (H16_A3288), which has been reported to be induced on trioleate [18], was moderately and constitutively expressed on fructose. H16_B1148, which encodes another ACS, was extremely induced in the PHA production phase. The cluster H16_A1067-A1070 was also induced in the PHA production phase. In particular, the induction ratio and expression levels of H16_A1067 and A1068, both encoding ACDH, were very high in F26. Both of H16_A1069 and A1070 were identified as genes that encode homologs of (*R*)-specific enoly-CoA hydratase (R-ECH), and the product of H16_A1069 (PhaJ4a) has been demonstrated to be an R-ECH that is specific to mcl-enoyl-CoAs [11]. These results strongly suggested that fatty acid β-oxidation was functional even in the presence of fructose in *R. eutropha* H16, and it may have a role in the active turnover of acyl moieties derived from lipids. Tsuge *et al*. reported that when *R. eutropha* PHB^-^4 expressed laboratory-evolved *phaC1* from *Pseudomonas* sp. 61-3, it accumulated PHA co-polyester which contained a small fraction of mcl-3-hydroxyalkanoate units from fructose [15]. It was assumed that the mcl-(*R*)-3-hydroxyacyl-CoA monomers were provided through the activated β-oxidation linked with lipid turnover when the cells were grown on fructose. The detection of the mcl-CoA-thioesters in *R. eutropha* H16 cells grown on fructose according to the metabolomic analysis [23] was consistent with this expectation.

#### P(3HB) biosynthesis

A previous quantitative-real time PCR (qRT-PCR) analysis, which used 16SrRNA as an internal quantification control, reveled that the relative expression levels of *phaC1-A-B1* (H16_A1437-A1439) decreased during the growth phase [36]. In contrast, a recent microarray analysis reported similar expression levels of *phaC1-A-B1* in conditions with or without a nitrogen source [22]. The RNA-seq analysis in the present study showed rather similar transcription levels of *phaA* and *phaB1*, as well as a 3.7-fold induction of *phaC1* expression in F26 when compared with F16. These contradictory results may have been caused by the use of different analytical platforms. Thus, we performed a detailed qRT-PCR analysis of *phaC1* using the total RNA samples prepared for RNA-seq with three primer sets (shown in Additional file 1: Table S4) and two inner controls (16SrRNA and *bfr2* [H16_A0328]). As shown in Additional file 1: Figure S1, when 16SrRNA was used as an inner control, the three amplifications of different *phaC1* regions indicated decrease of expression as longer cultivation time, which were in accordance with the previous qRT-PCR result [36]. However, qRT-PCR of *N*-terminal and central regions of *phaC1* with *bfr2* control indicated induction of the gene expression in the PHA production phase. It appeared that the induction behavior of *phaC1* was feasible, because the induced expression levels of *phaC1* in F26 based on qRT-PCR and RNA-seq agreed well with the strong positive correlation of the expression ratios of other genes obtained from different platforms, as shown in Additional file 1: Figure S2.

Of the 21 KT genes, *phaA*, *bktB* (H16_A1445), and H16_A0170 have been reported to be the major participants in P(3HB) biosynthesis [37]. The RNA-seq analysis revealed that the expression of *bktB* and H16_A0170 increased in the PHA production phase (Figure 3). In addition, we detected expression of other KT genes, namely, H16_A0462, H16_A1528, and H16_B0759 (Figure 4). This result coincided with the recent microarray analysis [22]. The former two genes are located within the β-oxidation clusters [18], which suggests the contribution of their gene products in thiolysis of medium/long-chain-length 3-ketoacyl-CoA intermediates during lipid turnover. Indeed, the disruption of H16_A1528 gave no effect on growth and PHB accumulation when grown on fructose [37]. The expression behaviors of *phaB2* (H16_A2002) and *phaB3* (H16_A2171), as well as the negligible transcription of the second PHA synthase gene *phaC2* (H16_A2003) were well agreed with the previous microarray analyses [17,22,38].

The PHA granule-associated proteins, which are known as phasins, are encoded by 7 genes in *R. eutropha* H16. *phaP1* (H16_A1381) encodes a major phasin, and its PHA biosynthesis-coupled induction was reported to be mediated by an autoregulator PhaR (H16_A1440) [39]. In our study, *phaP1* had the third highest expression level in F26 (Additional 1: Table S2). Pötter *et al*. proposed the PhaR-mediated regulation of *phaP3*[39], but the down-regulation of *phaP3* expression was observed in the PHA production phase. It was reported that PhaP3 was a major phasin in the *phaP1*-deficient mutant of *R. eutropha*[40]; therefore, the release of PhaR from the *phaP3* region may occur only in the absence of PhaP1. A previous observation suggested that PhaP2 (PHG202) was not present on the granule surface *in vivo*, whereas the expression level of *phaP2* was very high in the growth and PHA production phases. Another study suggested that PhaP2 may have indirectly participated in the formation of P(3HB) granule by interacting with other phasins [41]. In our study, *phaP4* (H16_B2021) was expressed during cultivation with the lower level than *phaP1* and *phaP2*. PhaP5 (H16_B1934) [41], PhaP6 (H16_B1988) and PhaP7 (H16_B2326) [42], and PhaM (H16_A0141) [43] were recently identified as new granule-associated proteins, although the expression levels of their corresponding genes were observed to be very low. The weak expression level of *phaP5* in F26 markedly contradicted with a previous microarray analysis [22]; hence, further validation will be necessary.

*R. eutropha* possesses 5 PHA depolymerases with a DepA domain (*phaZ1-Z5*), 2 additional depolymerases with an LpqC domain (*phaZ6* and *phaZ7*) and 2 hydroxybutyrate oligomer hydrolases (*phaY1* and *phaY2*) that are considered to be involved in mobilization of P(3HB). Despite the cellular phases examined in the present study were not the PHA utilization phase, the expression levels of *phaZ4* (PHG178) and *phaY2* (H16_A1335) in the growth phase; and *phaZ1* (H16_A1150) and *phaZ6* (H16_B2073) in the PHA production phase were rather higher than those of others.

#### Transporters

Kaddor *et al*. demonstrated that the fructose-specific ABC-type transporter FrcACB, which is encoded within the sugar degradation gene cluster 1, was essential for the growth of *R. eutropha* H16 on fructose [44]. We observed significant down-regulation of these genes in the PHA production phase compared with the growth phase, as described above (Figure 2 and Additional 1: Table S3). The weak expression level of *frcACB* may be sufficient to support an adequate carbon flux for PHA biosynthesis, or other transporters may have roles in this process. However, the resent microarray analysis reported up-regulation of the fructose transporter genes during nitrogen starvation [22]. *copP2* (H16_A3668), which encodes a putative copper uptake P-type ATPase; and *nosFD* (PHG249-PHG250), which encodes putative copper-specific ABC transporter subunits, were highly up-regulated in the growth phase along with *copDCBA* (H16_B2182-B2185) and *copZ* (H16_A3669) (Additional file 1: Table S3), which confer resistance to copper. The up-regulation of these genes was estimated to be due to formation of active copper-containing enzymes, such as cytochrome *c* oxidase, in an aerobic respiratory chain [45].

### ^13^CO_2_ Fixation into P(3HB) synthesized from fructose in the presence of NaH^13^CO_3_ by *R. eutropha* H16

It was not clear whether CBB cycle induced under the heterotrophic condition was actually functional in CO_2_ fixation; therefore, we examined the incorporation of ^13^C into P(3HB) synthesized by *R. eutropha* in the presence of NaH^13^CO_3_. First, the wild-type H16 strain was cultivated in a nutrient rich medium for cell growth, and P(3HB) biosynthesis was promoted in a nitrogen-free mineral salt medium that contained fructose with periodic additions of NaHCO_3_ (^12^C or ^13^C). It was confirmed that the cell growth was not occurring, but the P(3HB) content was increased from approximately 5 wt% to 50 wt% during the second stage. The abundance of ^13^C in the P(3HB) fraction after the addition of NaH^12^CO_3_ was determined to be 1.13% by gas chromatography–mass spectrometry analysis (GC-MS), which was the same as the natural ^13^C-abundance (Table 3). Notably, when NaH^13^CO_3_ was added to the medium, the abundance of ^13^C in P(3HB) increased to 2.22%. To elucidate the function of Rubisco(s) in ^13^CO_2_-fixation during the heterotrophic PHA production, we performed single and double deletions of the two sets of Rubisco genes [*cbbLS*_*c*_ (H16_B1394-B1395) in the *cbb*_*c*_ operon and *cbbLS*_*p*_ (PHG426-PHG427) in the *cbb*_*p*_ operon]. The recombinant strains were cultivated according to the same procedure and analyzed. The results showed that the abundance of ^13^C in P(3HB) was 1.25% within the double disruptant H16∆∆*cbbLS*. The slight increase from the natural ^13^C-abundance was assumed to be caused by anaplerotic carboxylation or other carboxylation reactions. The cultivation of another wild-type strain of *R. eutropha* JMP134, which lacks Rubisco and ribulose-5-phosphate kinase that are the two key enzymes in CBB cycle, also produced the same results as H16∆∆*cbbLS* (data not shown)*.* It was calculated that the wild-type H16 strain incorporated 8-fold more ^13^C into P(3HB) from NaH^13^CO_3_ when compared to H16∆∆*cbbLS*. The abundance of ^13^C- in P(3HB) synthesized by H16∆*cbbLS*_*c*_ and H16∆*cbbLS*_*p*_ were 1.81% and 2.11%, respectively, which were slightly lower than the abundance of ^13^C with H16 strain but higher than that with the double disruptant. Namely, both of the Rubiscos were involved in ^13^C-incorporation and were able to compensate for the lack of another enzyme to a considerable extent. The results indicated that, even in the heterotrophic condition on fructose, the transcriptionally activated CBB cycle was actually functional in CO_2_ fixation by *R. eutropha* H16. This was also supported by our recent detection of ribulose 1,5-bisphosphate, a key metabolite in CBB cycle, based on metabolomic analysis of *R. eutropha* H16 grown on fructose or octanoate [23].

**Table 3:** **Abundances of **^**13**^**C in P(3HB) synthesized by *R. eutropha *H16 and *cbbLS *disruptants on fructose with addition of NaH**^**13**^**CO**_**3**_^**a**^

***R. eutropha *strain**	**NaHCO**_**3 **_**added**^**b**^	**P(3HB) (wt%)**	^**13**^**C-Abundance in P(3HB)**^**c **^**(%)**	**Increase of **^**13**^**C in P(3HB) (mmol/g-P(3HB))**
H16	^12^C	53.6 ± 2.14	1.13 ± 0.0003	-
	^13^C	49.5 ± 4.39	2.22 ± 0.0025	0.42 ± 0.0016
H16∆*cbbLS*_*c*_	^12^C	52.1 ± 0.91	1.10 ± 0.0001	-
	^13^C	48.3 ± 1.41	1.81 ± 0.0013	0.27 ± 0.0007
H16∆*cbbLS*_*p*_	^12^C	50.0 ± 2.49	1.13 ± 0.0002	-
	^13^C	48.3 ± 2.48	2.11 ± 0.0022	0.38 ± 0.0012
H16∆∆*cbbLS*	^12^C	27.8 ± 0.17	1.11 ± 0.0003	-
	^13^C	30.0 ± 0.48	1.25 ± 0.0005	0.05 ± 0.0004

^a^P(3HB) biosynthesis was performed by 2-stage cultivation as described in the text.

^b^Added periodically during the second stage.

^c^Means of ^13^C/^12^C ratios calculated from isotopomer abundances of the three fragments (*m/z* 45, 87, and 103) derived from 3HB methyl ester.

## Conclusion

This study applied the RNA-seq technique to analyze the genome-wide transcriptional dynamics of PHA-producing *R. eutropha* H16. The mRNA enrichment using a commercially available probe specific to bacterial rRNA was incomplete for *R. eutropha* even after two repeated operations, but the greater depth of new sequencing technology could overcome this problem by giving sufficient numbers of reads from mRNA. A comparison of the transcriptomes detected several phase-depending changes in the expression of genes responsible for shifts in the physiological state of *R. eutropha* throughout cultivation on fructose. In the growth phase, there was high level induction of genes related to transcription, translation, cell division, peptidoglycan biosynthesis, pilus and flagella assembly, energy conservation, and fatty acid biosynthesis; while the genes related to central metabolism were repressed in the PHA production phase. Interestingly, the CBB cycle genes and several β-oxidation genes were transcriptionally activated in the PHA production phase compared with that in the growth phase, when fructose was supplied as the sole carbon source. We further found that ^13^CO_2_ was incorporated into P(3HB) when *R. eutropha* H16 was incubated in the fructose-containing medium in the presence of NaH^13^CO_3_. The incorporation of ^13^C was significantly reduced by the double disruption of both Rubisco genes, which demonstrated that the CO_2_ fixation was mediated by Rubisco, i.e., the transcriptionally activated CBB cycle was functional during heterotrophic PHA biosynthesis. To the best of our knowledge, this is the first report to demonstrate CO_2_ fixation into PHA under a heterotrophic condition. The results of our study will facilitate further metabolic engineering of *R. eutropha* for improved production of PHAs from non-fossil resources, such as the increased metabolic flux from sugars to PHA, the provision of mcl-(*R*)-3-hydroxyacyl-CoA monomers from sugars through lipid turnover, and fixation of CO_2_ into the polymer materials.

## Methods

### Cultivation, RNA isolation, and mRNA enrichment

*R. eutropha* wild strain H16 (DSM428) was cultivated in a 500 ml flask on a reciprocal shaker (115 strokes/min) at 30°C with 100 ml of a nitrogen-limited mineral salts (MB) medium, which was composed of 9 g/l Na_2_HPO_4_ · 12H_2_O, 1.5 g/l KH_2_PO_4_, 2.0 g/l NH_4_Cl, 0.2 g/l MgSO_4_ · 7H_2_O, and 1 ml/l trace element solution [46] in deionized water. A filter-sterilized solution of fructose was added to the medium at a final concentration of 20 g/L. When octanoate was used as a carbon source, 0.1% (w/v) of sodium octanoate (filter-sterilized) was added stepwise at 12 h intervals to avoid the toxic effects on cell growth.

The cells in 10 ml culture broth at 16, 26, and 36 h on fructose and 26 h on octanoate were harvested by centrifugation (1,400 *g*, 10 min, 4°C), and total RNA was isolated from the cell pellet by using RNeasy Midi Kit (Qiagen, Valencia, CA, USA). RNA eluted in 150 μl RNase-free water was treated with DNase I. 25–50 μg of the total RNA was then subjected to repeated treatment using RiboMinus Transcriptome Isolation Kit (Yeast and Bacteria) (Invitrogen, Carlsbad, CA, USA) for mRNA enrichment. Samples after the treatment were concentrated by ethanol precipitation and dissolved in 30 μl of RNase-free water. The removal of a large fraction of rRNA was confirmed by conventional agarose electrophoresis and ethidium bromide staining, and the quality and quantity of the enriched mRNA samples were assessed by 2100 Bioanalyzer (Agilent Technologies, Santa Clara, CA, USA).

### Library construction, sequencing, and data analysis

RNA-seq template libraries were constructed with 1 μg of the enriched mRNA samples using RNA-Seq Template Prep Kit (Illumina Inc., San Diego, CA, USA) according to the manufacturer's instructions. Deep sequencing was performed by Illumina GAIIx sequencer and 36 base-single end reads were generated.

The raw reads were mapped onto genome sequences of *R. eutropha* H16; NC_008313 (chromosome 1), NC_008314 (chromosome 2), NC_005241 (megaplasmid pHG1), using Burrows-Wheeler Aligner (BWA) [47]. The alignments with mismatch or mapped to the five rRNA regions of *R. eutropha* H16 (1806458–1811635, 3580380–3575211, and 3785717–3780548 on chromosome 1, and 174896–180063 and 867626–872793 on chromosome 2) were discarded, and the remaining reads were used as total reads. RPKM value (Reads Per Kilobase per Megabase of library size) [48] for each coding DNA sequence was calculated as a quantitative gene expression index by using custom Perl scripts. For multi-hit reads that did not aligned uniquely, the reciprocal number of the mapped loci was counted for the read. Analysis of variance (ANOVA) of the RPKM values obtained from the two replicates of the samples, and distributed visualization of the significantly changed genes in expression levels (*P* < 0.05) were performed by using MeV [49].

### PHA analysis

*R. eutropha* cells were harvested by centrifugation (5,000 *g*, 10 min, 4°C), washed with cold deionized water, centrifuged again, and then lyophilized. Cellular PHA contents were determined by gas chromatography (GC) after methanolysis of the dried cells in the presence of 15% (v/v) sulfuric acid in methanol, as described previously [46].

### Construction of disruption plasmids and strains

A plasmid pK18ms∆cbbLSc for deletion of *cbbLS*_*c*_ from chromosome 2 of *R. eutropha* H16 was constructed as below, and primers are listed in Additional file 1: Table S4. First, upstream and downstream regions (about 1 kbp) of *cbbLS*_*c*_ were individually amplified by PCR with genomic DNA of *R. eutropha* H16 as a template and primer sets of cbbLSc-up5’/cbbLSc-up3’ and cbbLSc-down5’/cbbLSc-down3’, respectively. The second PCR with the amplified fragments using cbbLSc-up5’/cbbLSc-down3’ primers gave a fused fragment of the upstream and downstream regions of *cbbLS*_*c*_. The resulting fragment was digested by EcoRI and HindIII and then ligated with pK18mobsacB [50] at the corresponding sites to obtain pK18ms∆cbbLSc. pK18ms∆cbbLSp for deletion of *cbbLS*_*p*_ from megaplasmid pHG1 was constructed in the same way using primer sets of cbbLSp-up5’/cbbLSp-up3’ and cbbLSp-down5’/cbbLSp-down3’.

Transconjugation of mobilizable plasmids from *E. coli* S17-1 to *R. eutropha* and isolation of strains generated by pop in-pop out recombination using the pK18mobsacB-based suicide plasmids were performed as described previously [13,14]. The strains H16∆*cbbLS*_*c*_, H16∆*cbbLS*_*p*_, and H16∆∆*cbbLS* were obtained by single deletion of *cbbLS*_*c*_ and *cbbLS*_*p*_, and double deletion of the genes in *R. eutropha* H16, respectively.

### Determination of the abundance of ^13^C in P(3HB)

Cultivation of *R. eutropha* strains H16, H16∆*cbbLS*_*c*_, H16∆*cbbLS*_*p*_, and H16∆∆*cbbLS* were done in a 500 ml flask on a reciprocal shaker (115 strokes/min) at 30°C. Firstly, the strains were cultivated in 100 ml of a nutrient rich medium composed of 10 g/l tryptone, 2 g/l yeast extract, and 1 g/l meat extract in tap water for 12 h. The grown cells in 50 ml of the culture broth were harvested, washed with a salt solution (9 g/l Na_2_HPO_4_ · 12H_2_O, 1.5 g/l KH_2_PO_4_ in deionized water), and then transferred into 100 ml of a nitrogen-free MB medium (pH6.5 adjusted with KH_2_PO_4_) containing 0.5% (w/v) fructose. The cells were further incubated for 24 h to promote P(3HB) biosynthesis. NaH^12^CO_3_ (1.08% ^13^C (natural abundance)) or NaH^13^CO_3_ (98% ^13^C) (Taiyo Nippon Sanso, Tokyo, Japan) was added to a final concentration of 5 mM periodically every 2.5 h during the second stage, taking into consideration loss of dissolved CO_2_ to the atmosphere.

The cells after the second stage cultivation were harvested, washed, and lyophilized as described above. The dried cells were subjected to methanolysis, and analyzed by GCMS-QC2010 system (Shimadzu, Kyoto, Japan) equipped with an InertCap 1 capillary column (ϕ0.25 mm, 30 m) (GL Science, Tokyo, Japan). ^13^C/^12^C ratios in the fragments of CH_3_–CH=OH^+^ (*m/z* 45), CH_3_–C(OH)H–CH_3_–C=O^+^ (*m/z* 87), and CH_3_–O–CO–CH_2_–CH=OH^+^ (*m/z* 103) derived from 3HB methyl ester were calculated from the respective isotopomer abundances, and the mean was referred as a abundance of ^13^C in the P(3HB) fraction.

### RNA-seq data accession number

The RNA-seq data used in this study have been deposited in the NCBI Gene Expression Omnibus (GEO) under the accession number of GSE47759.

## Competing interests

The authors declare that they have no competing interests.

## Authors’ contributions

RS carried out the experiments and data analyses, and wrote the manuscript. KC participated in the sample preparation and preliminary examination. YS carried out RNA-sequencing. IO and SN participated in the design and coordination of the study. TF designed the experiments and participated in the data processing and manuscript preparation. All authors read and approved the manuscript.

## Supplementary Material

Additional file 1 **Detection of phase-dependent transcriptomic changes and Rubisco-mediated CO**_**2 **_**fixation into poly(3-hydroxybutyrate) under heterotrophic condition in *Ralstonia eutropha *H16 based on RNA-seq and gene deletion analyses (Shimizu et al.). Figure S1.** Relative expression changes of *phaC1* determined by qRT-PCR using three primer sets for amplification and two inner control genes for quantification. Square, amplification of the central region (primers: phaC1-5’-Cent/phaC1-3’-Cent); diamond, amplification of the *N*-terminal region (phaC1-5’-N/phaC1-3’-N); circle, amplification of the *C*-terminal region (phaC1-5’-C/phaC1-3’-C). Open symbols, *bfr2* inner control; closed symbols, 16SrRNA inner control. Materials and Methods for qRT-PCR. **Figure S2.** Correlation of expression ratios from RNA-seq and qRT-PCR in F26. The best-fit linear regression curve is shown with the correlation coefficient (R^2^). Closed circle, *dapA1* (primers: dapA1-5’/dapA1-3’); closed square, *phaC1* (phaC1-5’-Cent/phaC1-3’-Cent); closed triangle, *cbbL* (cbbL-5’/cbbL-3’); closed diamond, *bfr2* (bfr2-5’/bfr2-3’). The primer sequences are listed in Table S4, and qRT-PCR was performed as described in the legend of Figure S1. **Table S1.** Highly transcribed genes in *R. euttopha* H16 during the growth on fructose.a. **Table S2.** Highly up-regulated genes in F26 to F16. **Table S3.** Highly down-regulated genes in F26 to F16. **Table S4.** Primers used in this study. Click here for file
