# Supplementary material for: Detection of phase-dependent transcriptomic changes and Rubisco-mediated CO2 fixation into poly (3-hydroxybutyrate) under heterotrophic condition in Ralstonia eutropha H16 based on RNA-seq and gene deletion analyses
Source: BMC Microbiol. 2013 Jul 23;13:169. doi: 10.1186/1471-2180-13-169 (PMC3734047; doi:10.1186/1471-2180-13-169)
Supplement: Additional file 1 — Detection of phase-dependent transcriptomic changes and Rubisco-mediated CO2 fixation into poly(3-hydroxybutyrate) under heterotrophic condition in Ralstonia eutropha H16 based on RNA-seq and gene deletion analyses (Shimizu et al.). Figure S1. Relative expression changes of phaC1 determined by qRT-PCR using three primer sets for amplification and two inner control genes for quantification. Square, amplification of the central region (primers: phaC1-5’-Cent/phaC1-3’-Cent); diamond, amplification of the N-terminal region (phaC1-5’-N/phaC1-3’-N); circle, amplification of the C-terminal region (phaC1-5’-C/phaC1-3’-C). Open symbols, bfr2 inner control; closed symbols, 16SrRNA inner control. Materials and Methods for qRT-PCR. Figure S2. Correlation of expression ratios from RNA-seq and qRT-PCR in F26. The best-fit linear regression curve is shown with the correlation coefficient (R2). Closed circle, dapA1 (primers: dapA1-5’/dapA1-3’); closed square, phaC1 (phaC1-5’-Cent/phaC1-3’-Cent); closed triangle, cbbL (cbbL-5’/cbbL-3’); closed diamond, bfr2 (bfr2-5’/bfr2-3’). The primer sequences are listed in Table S4, and qRT-PCR was performed as described in the legend of Figure S1. Table S1. Highly transcribed genes in R. euttopha H16 during the growth on fructose.a. Table S2. Highly up-regulated genes in F26 to F16. Table S3. Highly down-regulated genes in F26 to F16. Table S4. Primers used in this study. [file 1471-2180-13-169-S1.pdf]

Detection of phase-dependent transcriptomic changes and Rubisco-mediated CO_2 fixation into poly(3-hydroxybutyrate) under heterotrophic condition in *Ralstonia eutropha* H16 based on RNA-seq and gene deletion analyses (Shimizu et al.)

Supplementary information

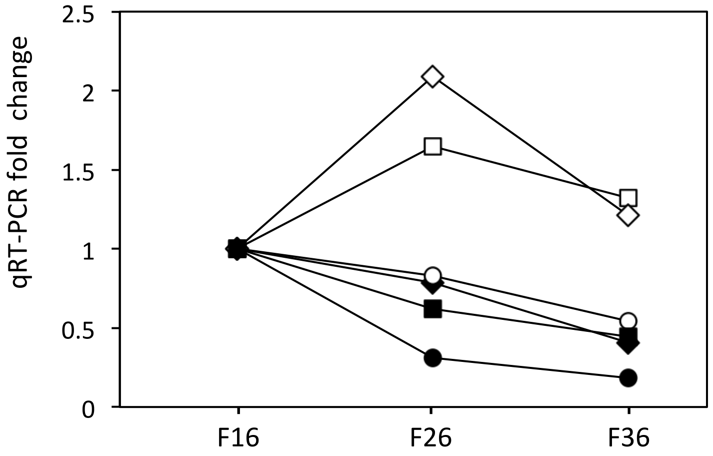

Fig. S1. Relative expression changes of *phaC1* determined by qRT-PCR using three primer sets for amplification and two inner control genes for quantification. Square, amplification of the central region (primers: phaC1-5'-Cent/phaC1-3'-Cent); diamond, amplification of the *N*-terminal region (phaC1-5'-N/phaC1-3'-N); circle, amplification of the *C*-terminal region (phaC1-5'-C/phaC1-3'-C). Open symbols, *bfr2* inner control; closed symbols, 16SrRNA inner control.

<u>Materials and Methods for qRT-PCR.</u>

The total RNA isolated for RNA-seq was used as templates. cDNA synthesis was performed using ReverTra Ace qPCR RT Master Mix (TOYOBO, Osaka, Japan) according to the manufacturer's recommendations. Primer sequences for real-time PCR were designed using Primer3 (http://frodo.wi.mit.edu/) and are listed in Table S4. Real-time PCR was performed using KOD SYBR qPCR Mix (TOYOBO, Osaka, Japan) with Thermal Cycler Dice Real Time System (Takara Bio, Otsu, Japan). The reaction conditions were: 2 min at 98 °C, 40 cycles × (10 s at 98 °C, 10 s at 60 °C, and 30 s at 68 °C). The gene expression levels of target regions were relatively quantified using following equation: Gene expression level=$2^{Ct(\text{Inner control})-Ct(\text{target region})}$.

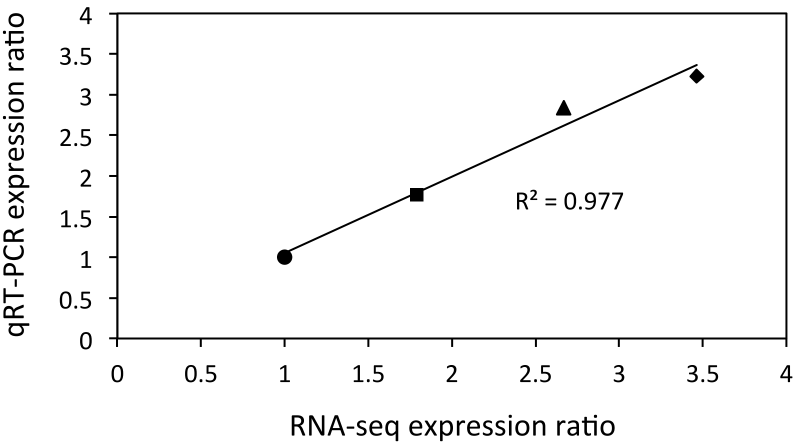

Fig. S2. Correlation of expression ratios from RNA-seq and qRT-PCR in F26. The best-fit linear regression curve is shown with the correlation coefficient (R^2). Closed circle, *dapA1* (primers: dapA1-5'/dapA1-3'); closed square, *phaC1* (phaC1-5'-Cent/phaC1-3'-Cent); closed triangle, *cbbL* (cbbL-5'/cbbL-3'); closed diamond, *bfr2* (bfr2-5'/bfr2-3'). The primer sequences are listed in Table S4, and qRT-PCR was performed as described in the legend of Fig. S1.

Table S1. Highly transcribed genes in *R. euttopha* H16 during the growth on fructose.[a]

Gene ID	Gene name	Product	F16	F26	F36	*P* value
H16_A0148		Hypothtical protein	3103	47027	23518	9.42E-6
H16_A0374	*ipk*	4-diphosphocytidyl-2-C-methyl-D-erythritol kinase	16166	23792	2798	4.66E-7
H16_A0386		Sigma54 (RpoN) modulation protein	22341	85644	51411	5.72E-4
H16_A0472		ABC transporter periplasmic protein	31321	7739	7906	2.22E-7
H16_A0482	*rplM*	50S ribosomal protein L13	60190	17355	4388	2.27E-7
H16_A0483	*rpsI*	30S ribosomal protein S9	52746	10359	5047	2.14E-8
H16_A0533		Universal stress protein	9457	20855	2059	6.41E-7
H16_A0705	*groES*	Co-chaperonin GroES	33882	10834	1032	2.93E-6
H16_A0706	*groEL*	Chaperonin GroEL	28093	6001	1324	3.62E-8
H16_A0788		Outer membrane protein or related peptidoglycan-associated (lipo)protein	26634	37691	7459	1.01E-6
H16_A0790		Hypothetical protein	23495	9595	6018	4.74E-7
H16_A0798	*rpsA*	30S ribosomal protein S1	45516	16769	8736	1.72E-7
H16_A0799	*ihfB*	Integration host factor subunit beta	27217	9100	10435	3.90E-6
H16_A0814	*fixA*	Electron transfer flavoprotein beta-subunit	10528	34930	3725	1.23E-8
H16_A0817	*dadA1*	D-amino acid dehydrogenase small subunit	3906	67927	22824	9.04E-8
H16_A0894	*rpsP*	30S ribosomal protein S16	22542	10196	3166	2.24E-6
H16_A0895	*rim*	16S rRNA-processing protein RimM	23590	6586	1772	1.24E-8
H16_A0896	*trmD*	tRNA (guanine-N(1)-)-methyltransferase	21765	4433	1556	5.77E-8
H16_A0897	*rplS*	50S ribosomal protein L19	89234	9919	7550	1.16E-7
H16_A0977		Hypothetical protein	32073	8589	1550	3.48E-7
H16_A1066		Hypothetical protein	2689	61243	1864	2.04E-9
H16_A1067		Acyl-CoA dehydrogenase	2264	29586	923	8.76E-8
H16_A1340	*infC*	Translation initiation factor 3	34741	26550	6823	1.35E-7
H16_A1341	*rpmI*	50S ribosomal protein L35	145428	39660	12481	1.88E-5
H16_A1342	*rplT*	50S ribosomal protein L20	145403	34556	14861	1.45E-7
H16_A1359		Hypothetical protein	13317	58595	8143	1.05E-7
H16_A1381	*phaP1*	Phasin (PHA-granule associated protein)	13550	78551	20363	7.99E-9
H16_A1439	*phaB1*	Acetyacetyl-CoA reductase	35196	24374	22457	2.06E-6
H16_A1482	*tig*	Trigger factor	21079	8960	2823	1.29E-9
H16_A2054	*tsf*	Elongation factor Ts	22557	2078	2299	7.06E-9
H16_A2276	*rplI*	50S ribosomal protein L9	42805	6470	4977	1.93E-9
H16_A2277	*rpsR*	30S ribosomal protein S18	40871	6261	4114	2.04E-6
H16_A2278		Primosomal replication protein N	24600	4662	2948	1.55E-8
H16_A2279	*rpsF*	30S ribosomal protein S6	38842	22167	5281	4.82E-8
H16_A2306	*infB*	Translation initiation factor IF-2	22829	4034	2692	1.30E-8
H16_A2307	*nusA*	Transcription elongation factor NusA	30394	8827	2475	6.63E-9
H16_A2308	*yhbC*	Hypothetical protein	19132	20280	1915	9.36E-8
H16_A2314		Hypothetical protein	20219	1742	894	3.24E-8
H16_A2354	*purA1*	Adenylosuccinate synthetase	8521	21641	5856	2.36E-8
H16_A2537		Hypothetical protein	8273	24129	159602	4.68E-6
H16_A2549	*efp2*	Elongation factor P	20069	13550	942	2.14E-6
H16_A2562	*rseA*	Negative regulator of sigma24 activity	8169	11758	31593	3.43E-7
H16_A2563	*rpoE1*	RNA polymerase sigma factor RpoE	9104	29706	26389	2.22E-7
H16_A2566	*acpP*	Acyl carrier protein	60340	22958	10760	4.47E-5
H16_A3035	*rpmG*	50S ribosomal protein L33	23353	13958	1929	2.33E-6
H16_A3053	*clpS*	ATP-dependent Clp protease adaptor protein ClpS	7543	29340	13070	7.51E-7
H16_A3178		Histone H1-like protein (alanine/lysin-rich protein)	44408	12231	17587	6.21E-7
H16_A3251	*rpmA*	50S ribosomal protein L27	62153	17279	8843	2.82E-8
H16_A3252	*rplU*	50S ribosomal protein L21	83506	31755	9204	6.33E-6
H16_A3268	*ftsZ*	Cell division protein FtsZ	15593	12244	25472	5.78E-6
H16_A3402		Outer membrane protein (porin)	200977	128814	28550	4.41E-7
H16_A3459	*rpsD*	30S ribosomal protein S4	21274	5308	1982	9.07E-8
H16_A3460	*rpsK*	30S ribosomal protein S11	28930	3613	4335	2.04E-10
H16_A3461	*rpsM*	30S ribosomal protein S13	38659	4179	4031	6.23E-7
H16_A3462	*rpmJ*	50S ribosomal protein L36	28941	3029	2529	5.69E-7
H16_A3463	*infA*	Translation initiation factor IF-1	38214	2466	2293	1.01E-5
H16_A3464	*secY*	Preprotein translocase subunit SecY	49712	2676	2801	5.48E-8
H16_A3465	*rplO*	50S ribosomal protein L15	42905	2713	3125	8.71E-7
H16_A3466	*rpmD*	50S ribosomal protein L30	46502	2721	2192	2.31E-7
H16_A3467	*rpsE*	30S ribosomal protein S5	77635	4196	3899	1.72E-7
H16_A3468	*rplR*	50S ribosomal protein L18	52464	3079	2766	2.74E-7
H16_A3469	*rplF*	50S ribosomal protein L6	81146	5593	3795	1.14E-8

H16_A3470	*rpsH*	30S ribosomal protein S8	76860	5740	3306	4.22E-9
H16_A3471	*rpsN*	30S ribosomal protein S14	63930	5362	3213	2.31E-7
H16_A3472	*rplE*	50S ribosomal protein L5	84150	5639	2772	5.26E-7
H16_A3473	*rplX*	50S ribosomal protein L24	88620	5517	3096	3.62E-8
H16_A3474	*rplN*	50S ribosomal protein L14	72917	6804	3129	1.11E-9
H16_A3475	*rpsQ*	30S ribosomal protein S17	62778	7206	7585	4.50E-6
H16_A3476	*rpmC*	50S ribosomal protein L29	63388	6417	6725	1.14E-6
H16_A3477	*rplP*	50S ribosomal protein L16	51564	5111	5606	5.69E-9
H16_A3478	*rpsC*	30S ribosomal protein S3	44982	4500	4747	2.02E-9
H16_A3479	*rplV*	50S ribosomal protein L22	57558	6548	5285	4.24E-8
H16_A3480	*rpsS*	30S ribosomal protein S19	48297	5655	3279	4.43E-8
H16_A3481	*rplB*	50S ribosomal protein L2	38478	5077	3613	2.41E-9
H16_A3482	*rplW*	50S ribosomal protein L23	24611	4157	1629	3.17E-8
H16_A3483	*rplD*	50S ribosomal protein L4	29386	5321	1823	7.03E-9
H16_A3484	*rplC*	50S ribosomal protein L3	45412	18305	2761	4.88E-9
H16_A3491	*Tufa*	Elongation factor Tu	56799	12188	3757	3.55E-8
H16_A3493	*rpsG*	30S ribosomal protein S7	48519	6689	4815	4.45E-7
H16_A3494	*rpsL*	30S ribosomal protein S12	29400	5059	2627	7.06E-8
H16_A3498	*rplL*	50S ribosomal protein L7/L12	97749	2926	4332	7.91E-8
H16_A3499	*rplJ*	50S ribosomal protein L10	117512	3245	5129	1.91E-9
H16_A3500	*rplA*	50S ribosomal protein L1	41872	2903	2637	7.27E-9
H16_A3501	*rplK*	50S ribosomal protein L11	41479	3140	2120	2.16E-6
H16_A3502	*nusG*	Transcription antitermination protein NusG	24253	4158	1684	2.64E-9
H16_A3503	*secE*	Preprotein translocase subunit SecE	22400	4491	912	1.11E-6
H16_A3505	*tufB*	Elongation factor Tu	69017	16690	4265	6.41E-9
H16_A3636	*atpC*	F_0F_1 ATP synthase subunit epsilon	22135	3990	755	1.18E-6
H16_A3637	*atpD*	F_0F_1 ATP synthase subunit beta	27006	4370	846	1.09E-10
H16_A3644	*atpI*	membrane-bound ATP synthase subunit, F_1-F_0-type proton-ATPase	12781	20053	901	4.27E-8
H16_A3684	*hupB2*	HU family DNA-binding protein	44354	8056	19061	7.17E-7
H16_B0002	-	Cold-shock protein, DNA-binding	31101	15436	45864	3.88E-4
H16_B0181	-	Fimbriae associated protein	36702	44610	19536	6.20E-6
H16_B0182	*cpaA2*	Flp pilus assembly protein, protease CpaA	20955	15717	4948	1.44E-6
H16_B0469	-	Hypothetical protein	198	31699	676	1.93E-6
H16_B0700	*prkA*	Serine protein kinase	1858	20900	71784	9.15E-6
H16_B0701	-	Hypothetical protein	1398	15296	73979	1.79E-8
H16_B1225	-	Hypothetical protein	1508	931	23583	5.60E-8
H16_B1381	-	Hypothetical protein	725	24092	727	1.00E-7
H16_B1581	*metE*	5-methyltetrahydropteroyltriglutamate-homocysteine S-methyltransferase	24722	9128	10651	1.17E-5
H16_B1672	-	XRE family-like protein	8381	11922	30035	2.45E-5
H16_B1724	-	Hypothetical protein	474	21652	9646	1.27E-6
H16_B1929	-	Cold shock protein, DNA-binding	9572	36143	3219	2.07E-7
H16_B2205	-	Cold shock protein, DNA-binding	125300	60528	53240	8.03E-5
H16_B2553	-	Hypothetical protein	6749	32666	6321	1.06E-7
H16_B2576	-	Hypothetical protein	1670	21884	5001	6.21E-7
H16_B2577	-	Hypothetical protein	2097	34186	8757	3.09E-8
PHG109	-	Putative ATPase	4493	25111	4353	7.79E-10
PHG197	-	Hypothetical protein	4098	26036	2372	3.97E-6
PHG200	*fhp*	Nitric oxide dioxygenase	25473	11576	1040	1.77E-6
PHG202	*phaP2*	Phasin	29279	29219	2217	2.09E-6
PHG228	-	Hypothetical protein	2662	39323	1120	1.39E-9

[a]The genes of which RPKM values were larger than 20,000 at least in one of the three phases on fructose were presented except for tRNA genes.

Table S2. Highly up-regulated genes in F26 to F16.[a]

Gene ID	Gene name	Product	F16	F26	LOG2 ratio (F26/F16)	*P* value
H16_A1954		Acyltransferase	18	5295	8.24	1.22E-7
H16_A1957		Hypothetical protein	32	7203	7.81	6.22E-10
H16_A1956		Carbon-nitrogen hydrolase	19	3973	7.71	2.35E-7
H16_A1950		Hypothetical protein	36	6884	7.58	7.02E-8
H16_A1955		Biotin synthase-related enzyme	28	5144	7.55	3.47E-9
H16_B0934	-	Hypothetical protein	112	19084	7.41	3.64E-9
H16_B0469	-	Hypothetical protein	198	31699	7.32	1.93E-6
H16_B0933	-	Hypothetical protein	66	10436	7.32	9.77E-6
H16_A1953		Selenophosphate synthetase-related protein	23	2727	6.92	2.33E-6
H16_A1951	*trkA2*	K+ transport flavoprotein	71	7228	6.67	3.46E-8
H16_A1949	*sbm2*	Methylmalonyl-CoA mutase	49	4198	6.44	1.39E-6
H16_B0776	-	Nitrate reductase [NADH]	58	4389	6.25	2.77E-6
H16_A0358		ABC transporter permease	32	1953	5.93	2.66E-9
H16_B0777	-	Flavoprotein FAD oxidoreductase	37	2225	5.93	1.37E-6
H16_A0357		ABC transporter ATPase	75	4508	5.92	5.58E-7
H16_A1596		Hypothetical protein	239	13315	5.80	8.73E-8
H16_B0779	*nasD*	Nitrite reductase, NAD(P)H-dependent, large subunit	55	3001	5.77	2.39E-7
H16_B0468	-	Putative arylsulfatase regulator	116	5969	5.69	4.65E-7
H16_B0780	*narK5*	Major facilitator superfamily transporter NNP family protein	24	1178	5.62	2.92E-8
H16_B1724	-	Hypothetical protein	474	21652	5.51	1.27E-6
H16_A2656		Hypothetical protein	67	2835	5.41	3.30E-5
H16_B1158	-	Hypothetical protein	101	4239	5.39	1.33E-5
H16_A1597		Hypothetical protein	114	4212	5.21	1.65E-7
H16_B0950	-	Hypothetical protein	60	2180	5.20	2.71E-7
H16_B2252	-	Hypothetical protein	140	5064	5.18	1.07E-6
H16_A2227	*iclB*	Isocitrate lyase	382	13129	5.10	1.05E-11
H16_B1381	-	Hypothetical protein	725	24092	5.05	1.00E-7
H16_B0326	-	LuxR family transcriptional regulator	149	4506	4.92	1.34E-6
H16_B2253	-	Hypothetical protein	169	4937	4.87	5.81E-6
H16_B1149	-	Hypothetical protein	101	2928	4.86	1.20E-5
H16_A0073		M20 family peptidase	228	6536	4.84	3.16E-6
H16_A1320		GntR family transcriptional regulator	162	4474	4.79	1.21E-7
H16_B1380	*cyaA*	AMP/GMP cyclase	79	2167	4.79	4.00E-8
H16_A0074		AraC family transcriptional regulator	77	2089	4.77	1.44E-5
H16_A2190		Hypothetical protein	180	4853	4.76	7.46E-5
H16_B1969	-	Hypothetical protein	123	3267	4.74	1.46E-5
H16_B0952	*ccmA2*	ABC transporter ATPase	58	1534	4.73	1.19E-8
H16_B0168	-	Putative multidrug resistance protein	74	1942	4.71	5.33E-6
H16_B0100	-	Hypothetical protein	169	4420	4.71	1.02E-4
H16_B1150	-	Hypothetical protein	117	2905	4.63	1.12E-5
H16_A1066		Hypothetical protein	2689	61243	4.51	2.04E-9
H16_B1161	-	TetR/AcrR family transcriptional regulator	538	12218	4.51	9.32E-9
H16_B1382	*clpC2*	ATP-dependent protease Clp, ATPase subunit	46	1032	4.50	5.96E-5
H16_B1190	-	3-hydroxyisobutyrate dehydrogenase	41	919	4.50	5.95E-4
H16_B0948	-	NAD(FAD)-dependent dehydrogenase	65	1456	4.50	3.93E-7
H16_B1189	*crt*	Enoyl-CoA hydratase	44	993	4.50	1.23E-5
H16_A0688		Amidinotransferase	163	3651	4.49	1.85E-4
H16_B0467	-	Hypothetical protein	135	3025	4.49	9.38E-8
H16_B0951	-	Hemoglobin-like protein (Hmp)	110	2447	4.48	2.74E-7
H16_A0959		Hypothetical protein	385	8558	4.48	2.96E-6
H16_B1968	-	Hypothetical protein	95	2089	4.47	8.71E-7
H16_B1028	*cyoD3*	bo3-type chinol oxidase, subunit IV	34	735	4.43	7.17E-6
H16_B0957	*ccmG2*	Thiol-disulfide isomerase involved in cytochromec biogenesis	73	1557	4.42	7.32E-4
H16_B1024	-	Major facilitator transporter	37	790	4.42	3.25E-5
H16_A1976	*copP1*	Putative copper uptake P-type ATPase	64	1303	4.35	7.63E-9
PHG153	-	Hypothetical protein	135	2683	4.31	2.45E-6
H16_B1156	-	Hypothetical protein	366	7226	4.30	1.34E-5
H16_A2801		MFS family transporter	107	2106	4.30	5.29E-6
H16_A0359		ABC transporter periplasmic protein	95	1859	4.29	9.28E-6
H16_B1027	*cyoC3*	bo3-type chinol oxidase, subunit III	41	798	4.28	8.10E-6
H16_B1536	-	Hypothetical protein	279	5315	4.25	1.20E-4
H16_A1853		AraC family transcriptional regulator	27	510	4.24	3.31E-5
H16_B1148	-	Acyl-CoA synthetase	188	3452	4.20	1.73E-7

H16_A2657		Metal-dependent hydrolase	65	1183	4.19	2.64E-6
H16_B1383	*cbbB*	Formate dehydrogenase, alpha chain	521	9385	4.17	2.66E-7
H16_B0775	-	Extra-cytoplasmic solute receptor	131	2331	4.16	3.26E-8
H16_A0689		Ornithine cyclodeaminase	237	4159	4.14	8.52E-5
H16_B2145	-	Hypothetical protein	168	2932	4.13	1.98E-4
H16_A1080	*ureD*	Urea amidohydrolase (urease) accessory protein UreD	265	4606	4.12	5.16E-6
H16_A0165		MerR family transcriptional regulator	3906	67927	4.12	9.04E-8
H16_A0817	*dadA1*	D-amino acid dehydrogenase small subunit	66	1138	4.12	5.61E-5
H16_B1431	-	Universal stress protein	95	1639	4.12	3.97E-7
H16_A2781		Epimerase, PhzC/PhzF-like protein	85	1465	4.12	1.62E-5
H16_B0060	-	Hypothetical protein	222	3835	4.11	2.14E-5
H16_A0164		Zn-dependent hydrolase including glyoxylase	357	6025	4.08	1.64E-6
H16_A3357	*acrA6*	Cation/multidrug efflux system, mebrane-fusion component	89	1469	4.05	1.26E-5
H16_B1549	-	Hypothetical protein	115	1895	4.05	3.26E-4
H16_B2577	-	Hypothetical protein	2097	34186	4.03	3.09E-8
H16_A2673		Hypothetical protein	1205	19472	4.01	1.22E-6
H16_A1079		ABC transporter ATPase	138	2205	4.00	1.77E-5

[a]The RNA levels comparison with F26 to F16 were log-transformed to base 2 and the genes which the value is over 4 were shown. The genes which RPKM value (average of two runs) is over 500 in F26 are listed.

Table S3. Highly down-regulated genes in F26 to F16.[a]

Gene ID	Gene name	Product	F16	F26	LOG2 ratio (F26/F16)	*P* value
H16_B0263	*flgD*	Flagellar basal body rod modification protein	850	6	-7.27	1.56E-6
H16_B2369	*fliG*	Flagellar motor switch protein G	614	6	-6.80	7.78E-7
H16_B1810	-	ABC transporter ATPase	1138	17	-6.11	3.55E-6
H16_B1499	*frcC*	ABC transporter permease	5862	90	-6.03	4.40E-7
H16_B0233	-	Methyl-accepting chemotaxis protein	2514	39	-6.03	4.48E-6
H16_A2384		Extra-cytoplasmic solute receptor	2568	44	-5.88	4.29E-6
H16_B2367	*fliE*	Flagellar hook-basal body complex protein	553	10	-5.79	7.03E-6
H16_B2183	*copC*	Copper resistance protein C	4817	91	-5.73	5.97E-6
PHG249	*nosF*	Putative copper ATP-binding ABC transporter	1038	20	-5.70	1.42E-7
H16_B2122	*tar2*	Methyl-accepting chemotaxis protein	1145	23	-5.67	6.14E-9
H16_A3669	*copZ*	Copper chaperone, heavy metal ion binding	8485	171	-5.64	5.59E-5
H16_B2182	*copD*	Copper resistance protein D, transmembrane component	5641	116	-5.61	2.70E-9
PHG250	*nosD*	Putative copper binding periplasmic protein	1804	38	-5.59	7.31E-6
H16_A2382		ABC transporter ATPase	1739	38	-5.52	6.87E-8
H16_B2184	*copB*	Copper resistance protein B, copper extruder	11823	259	-5.52	2.26E-7
H16_B0232	-	Methyl-accepting chemotaxis protein	1642	36	-5.51	3.35E-6
H16_B0268	*flgI*	Flagellar basal body P-ring protein	698	16	-5.49	1.96E-8
H16_B1809	-	ABC transporter permease	531	12	-5.47	5.75E-6
H16_B0244	*cheY1*	Chemotaxis response regulator	1503	35	-5.44	7.33E-7
PHG334	*pri*	Putative primase	700	17	-5.41	2.10E-4
PHG349	*pilO*	Putative component of type II secretion apparatus	750	18	-5.38	1.99E-5
H16_B1501	*zwf2*	Glucose-6-phosphate 1-dehydrogenase	2828	75	-5.25	3.17E-7
H16_B1502	*pgi*	Glucose-6-phosphate isomerase	4466	123	-5.18	2.16E-5
H16_A3499	*rplJ*	50S ribosomal protein L10	2101	58	-5.18	9.04E-7
PHG289	-	Hypothetical protein	117512	3245	-5.18	1.91E-9
H16_A2381		ABC transporter permease	3442	97	-5.16	1.91E-7
H16_B1811	-	Hypothetical protein	1099	31	-5.15	1.04E-5
PHG288	-	Hypothetical protein	794	23	-5.14	9.23E-8
H16_B0234	-	Hypothetical protein	1855	54	-5.12	4.30E-6
H16_B0271	*flgL*	Flagellar hook-associated protein FlgL	1005	30	-5.07	2.88E-8
H16_A3498	*rplL*	50S ribosomal protein L7/L12	97749	2926	-5.06	7.91E-8
H16_B2181	-	Hypothetical protein	6095	186	-5.04	1.22E-7
H16_A0040		Hypothetical protein	638	20	-5.03	6.65E-4
H16_B0565	*fliN*	Flagellar motor switch protein/type III secretory pathway protein	1068	34	-4.99	2.72E-5
PHG348	*pilQ*	Putative traffic warden ATPase	715	24	-4.93	1.81E-5
PHG351	*pilN*	Putative component of type II secretion apparatus	582	20	-4.90	1.66E-7
H16_B1933	-	Methyl-accepting chemotaxis protein	3871	134	-4.86	5.13E-6
H16_B1498	*frcA*	ABC transporter ATPase	1156	40	-4.85	2.41E-7
H16_B1500	*frcB*	ABC transporter periplasmic protein	2713	98	-4.80	5.84E-6
H16_B0243	*cheB1*	Chemotaxis-specific methylesterase	635	23	-4.79	1.04E-5
H16_B0264	*flgE*	Flagellar hook protein FlgE	902	33	-4.77	5.94E-6
H16_A3602	*pyk2*	Pyruvate kinase	711	27	-4.72	9.50E-6
H16_A2385	*hmgL1*	Hydroxymethylglutaryl-CoA lyase	1815	69	-4.72	2.29E-4
H16_A3668	*copP2*	Putative copper uptake P-type ATPase	8340	320	-4.70	1.56E-5
H16_B1503	*frcK*	Fructokinase	3304	131	-4.66	2.19E-5
H16_B2185	*copA*	Copper resistance protein A, multi-copper oxidase	10580	427	-4.63	2.12E-6
PHG251	*nosR*	Putative transcription regulator	1400	59	-4.57	1.79E-7
H16_B2362	*fliD*	Flagellar hook-associated protein 2	1600	70	-4.51	2.86E-8
H16_B0231	*cheW3*	Chemotaxis protein	1203	57	-4.40	1.09E-5
H16_A2380		Signal transduction histidine kinase	6439	311	-4.37	1.14E-9
H16_B2360	*fliC*	Flagellin	549	27	-4.37	4.72E-6
H16_A3384		Hypothetical protein	1659	83	-4.32	5.76E-6
H16_B0245	*cheZ1*	Chemotaxis regulator CheZ	549	28	-4.32	1.61E-5
H16_B0270	*flgK*	Flagellar hook-associated protein FlgK	539	27	-4.32	1.23E-5
PHG355	*trbN*	Putative lytic transglycosylase	774	39	-4.31	4.19E-7
PHG292	-	Hypothetical protein	1673	86	-4.29	1.01E-5
PHG350	-	Hypothetical protein	542	28	-4.27	2.67E-5
H16_B2180	-	Hypothetical protein	2501	132	-4.25	1.36E-4
H16_B0258	*flgN*	Flagellar biosynthesis protein	547	29	-4.24	3.24E-7
PHG356	*trbI*	Putative channel-forming protein	1121	60	-4.24	1.98E-7
H16_B0239	*cheA1*	Chemotaxis protein histidine kinase	1140	61	-4.22	1.21E-8
H16_A2383		ABC-type transporter, periplasmic component	49712	2676	-4.22	5.48E-8
H16_A3464	*secY*	Preprotein translocase subunit SecY	4727	255	-4.22	1.66E-6

H16_A3467	*rpsE*	30S ribosomal protein S5	77635	4196	-4.21	1.72E-7
H16_A1377	*pdhL*	Dihydrolipoamide dehydrogenase (E3) component ofpyruvate dehydrogenase	15507	849	-4.19	5.50E-8
H16_B2434	-	Hypothetical protein	5194	291	-4.16	7.90E-6
H16_A3466	*rpmD*	50S ribosomal protein L30	46502	2721	-4.10	2.31E-7
H16_A3468	*rplR*	50S ribosomal protein L18	52464	3079	-4.09	2.74E-7
H16_B0240	*cheW1*	Chemotaxis protein	1595	96	-4.06	1.93E-5
H16_A1376	*hslV*	ATP-dependent protease HslVU (ClpYQ), peptidase subunit	12023	731	-4.04	1.77E-6
PHG352	*bfpG*	Putative BfpG-like protein	539	33	-4.03	2.36E-4
H16_A3473	*rplX*	50S ribosomal protein L24	88620	5517	-4.01	3.62E-8

[a]The RNA levels comparison with F26 to F16 were log-transformed to base 2 and the genes which the value is under -4 were shown. The genes which RPKM value (average or two run) is over 500 in F16 were listed.

Table S4. Primers used in this study.

Primer	Sequence (5'-3')	Restriction site[a] or additional information
cbbLSc-up5'	CCCAAGCTTATCAGCCCCCCGTACTCCCTC	HindIII
cbbLSc-up3'	ACTCGAAAGAAGCCCACCCACATAAGGTAAA	
cbbLSc-down5'	TGGGTGGGCTTCTTTCGAGTCGGATGCT	
cbbLSc-down3'	GGAATTCGATGGTGGTCAGGGTATGGT	EcoRI
cbbLSp-up5'	CCCAAGCTTTATTCCGCCGTGTGTTCCA	HindIII
cbbLSp-up3'	GTCAGGAAATCTGCGTTGATGCAAAGATA	
cbbLSp-down5'	ATCAACGCAGATTTCCTGACTGTGCCATCC	
cbbLSp-down3'	GGAATTCCAGGTGAGCGGTTTCGGTAT	EcoRI
phaC1-5'-Cent	GTGGTGGAGCAGGGACATAC	For amplification of 793-953 region of *phaC1*
phaC1-3'-Cent	AAGCCGAGCACGTTGATCTT	
phaC1-5'-N	CCGCGTTCTACCTGCTCAA	For amplification of 350-576 region of *phaC1*
phaC1-3'-N	GCGTGTCAGGTCTTCCATCA	
phaC1-5'-C	CGTTCGACCTGCTGTTCTGG	For amplification of 1256-1455 region of *phaC1*
phaC1-3'-C	CCACGGCACGATATGGTCTT	
16SrRNA-5'	CGGAATTACTGGGCGTAAAG	Ref[b]
16SrRNA-3'	ACGCATTTCACTGCTACACG	
bfr2-5'	CGCGATCAACCAGTATTTCC	
bfr2-3'	GTCTGCTCCAGCTTCAGGTC	
cbbLc-5'	CGCTTCCTGTTCGTGATGG	
cbbLc-3'	GGTGCAGGATCATGTCGTTCT	
dapA1-5'	CGACCCAGGAAGGCATGTA	
dapA1-3'	GCCGCTGTAGATGGCAAA	

[a] Indicated by underlining in the primer sequence.

[b] Lawrence AG, Schoenheit J, He A, Tian J, Liu P, Stubbe J, Sinskey AJ: Transcriptional analysis of *Ralstonia eutropha* genes related to poly-(*R*)-3-hydroxybutyrate homeostasis during batch fermentation. *Appl Microbiol Biotechnol* 2005, 68:663–672.
